# Anomalous Light Scattering by Topological *PT*-symmetric Particle Arrays

**DOI:** 10.1038/srep38049

**Published:** 2016-12-01

**Authors:** C. W. Ling, Ka Hei Choi, T. C. Mok, Zhao-Qing Zhang, Kin Hung Fung

**Affiliations:** 1 Department of Applied Physics, The Hong Kong Polytechnic University, Hong Kong, China; 2 Department of Physics, The Hong Kong University of Science and Technology, Hong Kong, China

## Abstract

Robust topological edge modes may evolve into complex-frequency modes when a physical system becomes non-Hermitian. We show that, while having negligible forward optical extinction cross section, a conjugate pair of such complex topological edge modes in a non-Hermitian 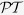-symmetric system can give rise to an anomalous sideway scattering when they are simultaneously excited by a plane wave. We propose a realization of such scattering state in a linear array of subwavelength resonators coated with gain media. The prediction is based on an analytical two-band model and verified by rigorous numerical simulation using multiple-multipole scattering theory. The result suggests an extreme situation where leakage of classical information is unnoticeable to the transmitter and the receiver when such a 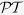-symmetric unit is inserted into the communication channel.

Parity-time (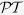)-symmetric quantum mechanics^1^ has opened up a new direction in searching for unconventional states of matter. Non-Hermitian 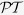-symmetric systems have been a subject of intense studies because they exhibit unusual properties, such as 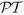-phase transitions^2^. While realization in quantum systems could be in doubt, several unconventional 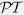-symmetry related phenomena, such as transition from real-frequency modes to conjugate pair of complex-frequency modes, have been applied in classical photonic systems^2^,^3^,^4^,^5^,^6^,^7^,^8^,^9^,^10^,^11^,^12^. Recently, many efforts have been put on extending topological band theory^13^,^14^,^15^,^16^,^17^,^18^,^19^,^20^,^21^ to non-Hermitian 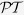-symmetric systems. For example, there have been different theoretical approaches to generalize topological invariants using bi-orthonormal basis^22^,^23^,^24^,^25^, redefining the inner product^26^, or using the global Berry phase^27^. Topological transition in the bulk of non-Hermitian system has also been realized^28^. It has been proposed that eigenstates of these non-Hermitian system associated with exceptional points could lead to new physics and applications, such as realization of Majorana zero modes^29^,^30^ and single mode lasers^7^,^8^,^31^.

Topological edge modes may evolve into complex-frequency modes when the system becomes non-Hermitian^32^,^33^,^34^. The physical consequence of such complex topological edge modes, which decay in both space and time, is obscure so far. In this paper, we suggest a way to realize complex-frequency topological edge modes in 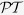-symmetric photonic systems. We show that a conjugate pair of topological complex-frequency edge modes in a 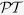-symmetric photonic systems can be realized through observing an anomalous sideway scattering by an array of subwavelength resonators coated with gain media. This suggests an extreme situation where leakage of classical information is unnoticeable to the transmitter and the receiver when such a 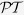-symmetric unit is inserted to the communication channel.

## Two-band 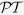-symmetric model

We begin with the topological description of a non-Hermitian 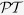-symmetric periodic system. It can be shown that, even if the edge mode frequencies become complex in non-Hermitian 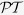
- symmetric systems, the Zak geometrical phase of a bulk band is still quantized as 0 or *π* when all bulk modes are in the unbroken 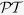-symmetric regime. To provide a complete description and proof, we start by considering a two-band, non-Hermitian 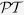-symmetric model which can be used to describe an array of subwavelength photonic resonators coated with gain media. The 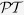-symmetric eigenvalue problem is written in the generic form as^27^,^35^


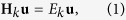


where


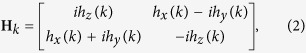


*E*_*k*_ is the eigenvalue, **u** is the right eigenvector, *k* is the Bloch wave vector, and *d* is the lattice constant.

**Figure 1:**
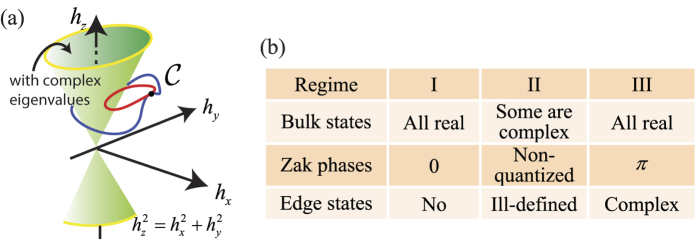
Classification of topological bands and edge modes of a 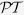-symmetric system described by Eq. (1). (**a**) Two types of paths in parameter space. The blue and the red path have winding number 1 and 0, respectively. (**b**) Table summarizing the three regimes according to the bulk and the edge state frequencies, including the Zak phase. In regimes I and III, the bulk states have real eigenvalues and the entire bulk dispersion relation is in the unbroken 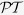-symmetric phase. In regime II, some bulk states are associated with complex eigenvalues and thus part of the bulk states are in the broken 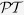-symmetric phase.

It should be noted that *h*_*x*_(*k*), *h*_*y*_(*k*), and *h*_*z*_(*k*) are periodic real-valued functions with period of 2*π*/*d* in *k*, and they form a 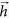-vector space as shown in Fig. 1(a). The subscripts *x, y*, and *z* refer to the directions in the 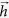-vector space (not the real space), while the expressions are determined by the actual approximation. Each system is represented as a closed loop 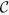 in Fig. 1(a) as the parameter *k* varies from −*π*/*d* to *π*/*d*. This closed loop is associated with a winding number which determines the Zak geometric phase of a band.

The Zak phase of each band (+ or −) is usually expressed as^18^,^20^,^21^,^36^,^37^,^38^,^39^


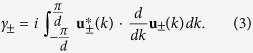


The analytical evaluation of Zak phase is given in the Methods. When *E*_*k*±_ are real for all *k* ∈ [−*π*/*d, π*/*d*] (i.e., all bulk states in the unbroken 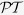-symmetric regime), the whole loop 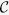 is outside the kissing cones 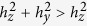 [see Fig. 1(a)]. Under this condition, we have quantized Zak phase


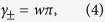


where *w* is the (integer) winding number of 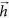
(*k*) about the *h*_*z*_ axis as *k* varies. It can also be shown that all even (odd) integer values of *w* are equivalent and Zak phase is thus usually quantized as zero or *π*. Since there exist different approaches in defining the Zak phase, we also provide a comparison to the results in bi-orthonormal basis (see Supplementary Information).

Zak phase can be used to classify the bulk band topology due to gauge invariance. More importantly, its value can help us predict the existence of the edge modes when all bulk states are in the unbroken 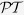-symmetric phase. For a large but finite 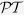-symmetric system, the weakly coupled edge modes also guarantee the existence in form of conjugate pair. In this case, the system can be classified (according to the bulk states) as three regimes summarized in Fig. 1(b). In this paper, we focus on the complex edge modes in regime III which can be simultaneously excited by a plane wave. In general, when 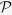 symmetry is broken, a linear combination of the edge modes can be excited so that the symmetry of the scattered waves may not follow the spatial symmetry of the plane wave. In the next section, we use a concrete example to show that when parity (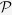) and time-reversal (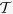) symmetries are broken but 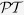 symmetry is not broken, the spatial symmetry of the scattered waves can be nearly opposite to that of the excitation plane wave, which finally gives rise to a negligible forward optical extinction but non-zero sideway scattering.

## 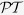-symmetric plasmonic particle array

**Figure 2:**
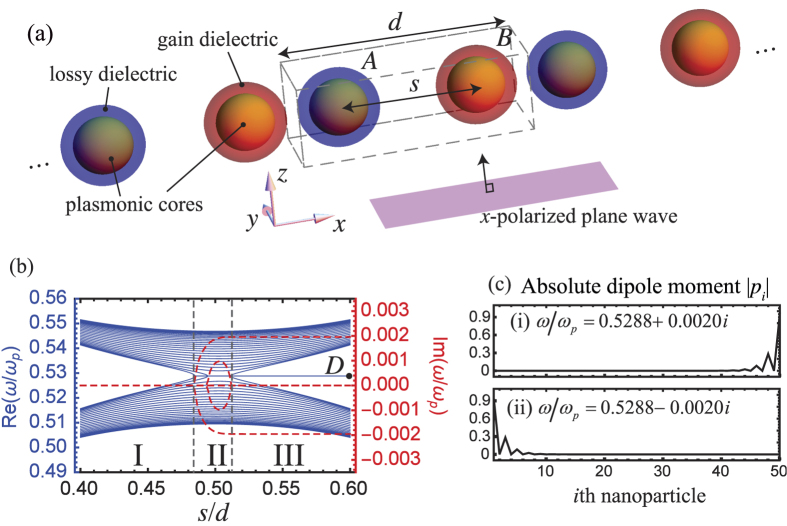
Eigenmodes of the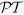-symmetric plasmonic particle array calculated based on coupled dipole method. (**a**) Schematic of the 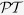-symmetric plasmonic particle array, which is an array of alternatively coated nanoparticles. The coated nanoparticles have inner radius *a* and outer radius *b*. A unit cell contains nanoparticle *A* and *B* with identical sizes while their shells are lossy and gain dielectrics with *ε*_3_ and 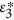 as the dielectric constants (gain-loss-balanced). In (**b**) and (**c**), we set *a* = 0.125*d, b* = 0.175*d*, and ε_3_ = 1.5 + 0.025*i* for the plasmonic particle array. (**b**) Resonant frequencies *ω* of a finite array (25 unit cells) against *s*. The bands are classified into three regimes (I to III). The blue horizontal line in regime III is associated with the dashed lines at Im(*ω*/*ω*_*p*_) = ± 0.002, which corresponds to a pair of topological edge modes. (**c**) Dipole moment patterns (absolute value) of the topological edge modes at point *D* in (**b**), where *s* = 0.6*d*. (Note that when *s* = 0.5*d*, nanoparticles are equally separated x).

We consider a spatially periodic plasmonic structure in which the electromagnetic modes are represented by the eigenvalue problem equivalent to Eq. (1) and takes the form similar to Bergman’s representation^40^,^41^,^42^,^43^,^44^,^45^,^46^:


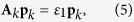


where *k*, **p**_*k*_, and *ε*_1_ are the Bloch wave vector, eigenvector, and eigenvalue respectively. The problem corresponds to a dimer array formed by two types of coated spherical plasmonic nanoparticles with alternative separations, *s* and *d* − *s*, as shown in Fig. 2(a). The coating shells are non-dispersive dielectrics, and are gain-loss-balanced. It should be noted that there have been previous studies on plasmonic 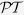-symmetric systems^47^,^48^. Here, we focus on the topological edge modes^20^.

Eq. (5) comes from the coupled dipole equation^20^,^45^,^49^,^50^, which is obtained by approximating each nanoparticle as a point dipole scatter, using the Bloch’s theorem, and taking the quasistatic approximation (see Methods). The dipole moments on nanoparticle *A* and *B* are denoted by the vector **p**_*k*_ = (*p*_*k*;*A*_, *p*_*k*;*B*_)^T^, while the matrix **A**_*k*_ is related to dipole couplings and the polarizabilties. Eigenvalue *ε*_1_ is also the dielectric function of the plasmonic core, and is mapped to the plasmon frequency *ω* via the Drude model^20^,^45^,^49^,^50^, given by


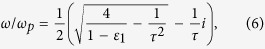


in which *ω*_*p*_ and 1/*τ* are the plasma frequency and electron collision frequency for the plasmonic core.

The finite plasmonic particle array has no 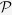 and 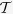 symmetries but 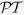-symmetry when the plasmonic cores are lossless (1/*τ* = 0). A detailed discussion on symmetry operators is given in the Methods.

## Complex edge modes

The longitudinal mode (*x* component) frequencies of a finite 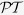-symmetric particle array when 1/**τ** = 0 are shown in Fig. 2(b). We focus on the longitudinal oscillation since it only weakly couples with the light line. As described by the table in Fig. 1(b), there are three regimes (I to III) in Fig. 2(b). In regime I, two bands can be clearly identified, and all *ω* are real. This implies all the eigenvalues *ε*_1_ are real, so the entire bulk dispersion relation is in unbroken 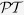-symmetric phase. Regime III is similar to regime I except that there are two complex topological edge modes supported in the band gap in regime III. The frequencies of these two topological edge modes form a complex conjugate pair, which is associated with the horizontal blue line and the red lines at Im(*ω*/*ω*_*p*_) = ±0.002. The existence of these complex edge modes are due to *γ* = *π*, which is classified as the (non-trivial) regime III. The evaluation of Zak phase for different gain/loss parameters are provided in Supplementary Information.

The two edge modes are eigenmodes with simultaneous complex frequency and complex wavevector, meaning that they decay or grow in both space and time. If the array is long enough, we have the following closed form solution (see Methods):


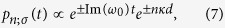


where *κ* = (3/*d*)ln[*s*/(*d* − *s*)], *ω*_0_ is the complex frequency of an edge mode, and *p*_*n*;*σ*_(*t*) represents the time-domain dipole moments in the *n*th unit cell. Both of the edge modes decay spatially, and the mode patterns at point *D* are shown in Fig. 2(c) for reference. Although the relation between spatially decaying modes and broken time-reversal symmetry has been studied^51^, the physical consequence of modes that decay in both space and time is obscure. In the next section, we will see that these modes can give rise to an anomalous scattering phenomenon.

## Vanishing optical extinction cross section at resonance


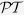
-symmetric particles and arrays have been shown to support very interesting properties^52^,^53^,^54^,^55^,^56^,^57^. Here, we study an unusual zero optical extinction in a situation where the topological complex modes are excited. To excite the topological edge modes in regime III, we introduce a *x*-polarized electrical plane wave with magnitude *E*_0_ and frequency *ω*, as shown in Fig. 2(a). The excited frequency-domain dipole moments on the 2*N* nanoparticles **p** = (*p*_1_, *p*_2_, …, *p*_2*N*_)^T^ are linearly dependent on the external wave **E**_0_ = (*E*_0_, *E*_0_, …, *E*_0_)^T^ (see Methods):


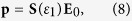


where *S(ε*_1_) is a square matrix depending on *ε*_1_, and explicit expression is shown in Eq. (21).

**Figure 3:**
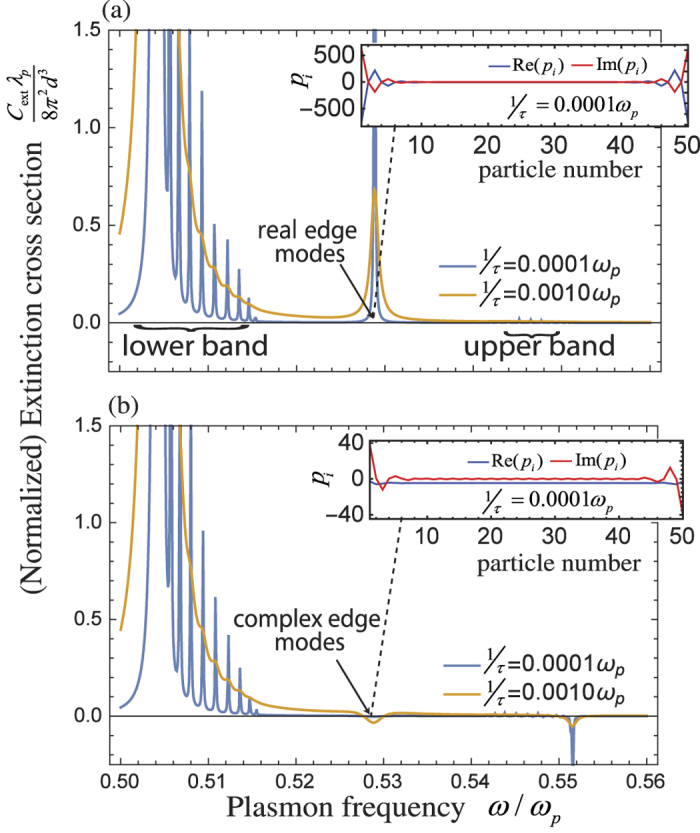
Comparison of optical extinction cross sections by plane waves between (**a**) a normal particle array (*ε*_3_ = 1.5) and (**b**) a 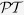-symmetric particle array (*ε*_3_ = 1.5 + 0.025*i*) with inner separation *s* = 0.6*d. λ*_*p*_ = 2*πc*/*ω*_*p*_. The extinctions dropped at *ω* = 0.529*ω*_*p*_ with small finite Im(*ε*_3_). The embedded subfigures are the excited dipole moments *p*_*i*_ in each case at *ω* = 0.529*ω*_*p*_ and external wave with electric field magnitude *E*_0_ = 1. Other parameters are *s* = 0.6*d, a* = 0.125*d, b* = 0.175*d*, and *N* = 25.

In Fig. 3, we show the corresponding extinction cross section calculated in dipole approximation^58^,^59^


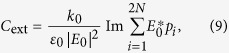


where *k*_0_ = *ω*/*c, c*, and *ε*_0_ are, respectively, the photon wavenumber, the speed of light, and the permittivity in free space.

In Fig. 3(a), the peak at *ω* = 0.529*ω*_*p*_ is due to the conjugate pair of edge modes, which drops drastically when Im(*ε*_3_) slightly increases from 0 [Fig. 3(a)] to 0.025 [Fig. 3(b)]. Surprisingly, the two edge modes are strongly excited in Fig. 3(b) (as shown by the normalized dipole moment in the inset) while the corresponding optical extinction cross section is vanishing.

To explain the vanishing *C*_ext_ in Fig. 3(b), we define and evaluate the symmetry operators (see Methods for details). The time reverse operator 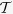 turns *p*_*i*_ into its complex conjugate (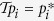). On the other hand, the inversion operator 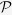 flips the direction of *p*_*i*_ and reverse the position order of the nanoparticles (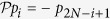). Consequently, their combination, transforms a column vector *p*_*i*_ and a general 2*N* × 2*N* matrix *A*_*i, j*_ in the way that 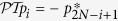. Since the matrices **A** and **S**(*ε*_1_) commute with 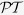 when *ε*_1_ is real (see Methods for a proof), we have 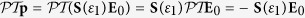 and thus


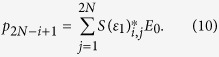


when the incident field is uniform (i.e., *E*_0*i*_ = *E*_0_). The result is consistent with the actual dipole moments (according to Eq. (8)) as shown in the insets of Fig. 3(b) by setting *E*_0_ = 1. At the frequencies when only complex eigenmodes are excited (i.e., no divergence of extinction due to real eigenmode), we have


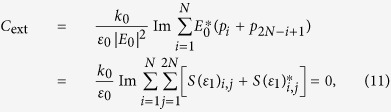


which means that the simultaneously excited edge modes give vanishing *C*_ext_ in the perfect 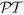 situation.

It should be noted that when real eigenmodes are excited, the *C*_ext_ should normally diverge at the resonance frequencies. In the case of complex eigenmode being excited, the *C*_ext_ would be finite. In discussion above, we conclude that extinction cross section is vanishing even if the complex topological edge modes are excited. Due to finite absorption and radiation effects, we found that the net extinction cross section could be below zero, which means that there is net finite amount of energy emitted from the array of particles. As shown in Fig. 3(b), it is clear that an anti-symmetric response of the array is shown, which will contribute to net radiation but the field is orthogonal to the plane wave such that it does not contribute to forward or backward scattering along the propagating direction of incident wave.

## Verifications by multiple scattering theory

**Figure 4:**
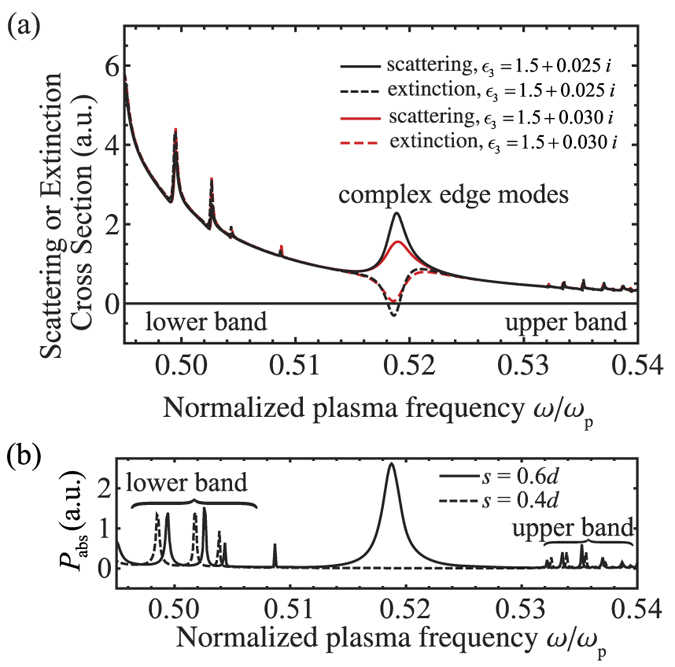
Verification by MST with the contribution from multipoles up to the 5th order. (**a**) Scattering and extinction cross sections of 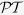-symmetric particle arrays with ε_3_ = 1.5 + 0.025*i* and 1.5 + 0.03*i (s* = 0.6*d*). (**b**) Integrated field intensity *P*_*abs*_ of the mostly excited particle when *ε*_3_ = 1.5 + 0.025*i*, where *P*_*abs*_ is proportional to *ω*∫|**E**(**r**)|^2^*d*^3^*r* over the particle volume. The dashed peak in (**b**) at *ω* = 0.5188*ω*_*p*_ indicated that the edge modes are strongly excited simultaneously. Other parameters are *a* = 0.125*d, b* = 0.175*d, d* = 100nm, *N* = 16, and 1/*τ* = 0.0001*ω*_*p*_.

**Figure 5:**
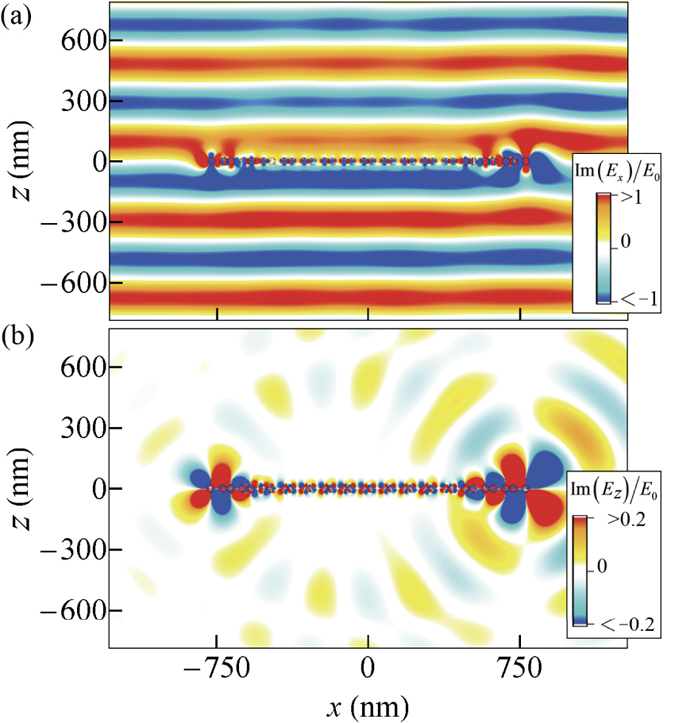
Electric field patterns at *ω* = 0.5188*ω*_p_ of the 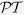-symmetric particle array (ε_3_ = 1.5 + 0.025i) demonstrated in Fig. 4(a). Panel (a) and (b) display the *x* and the *z* components of the field. The real part of *E*_*z*_ component is not shown for simplicity since it is dominated by the imaginary part. A symmetric distribution of *E*_*z*_ means an anti-symmetric distribution of dipole moments. Due to anti-symmetric resonance response, the array gives strong off-normal scattering but weak distortion to the (forward) transmission and (backward) reflection. Other parameters are *a* = 0.125*d, b* = 0.175*d, d* = 100nm, *N* = 16, and 1/*τ* = 0.0001*ω*_*p*_.

We present the verifications by Multiple Scattering Theory (MST)^60^,^61^ which includes up to the 5th order multipoles as well as dynamic interactions in Figs 4 and 5. The scattering and extinction cross sections of 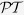-symmetric particle arrays calculated by MST are shown in Fig. 4. Remarkably, it is shown in Figs 4 and 5 that the simultaneously excited edge modes (*ω* ~ 0.52*ω*_*p*_) provide strong scattering cross section but negligible forward extinction. This is due to the net emission of energy associated with the slightly imbalance between gain and loss since there exists additional radiation loss in realistic situation beyond the quasi-static dipole approximation in previous sections.

Although the forward extinction is nearly zero due to anti-symmetric response, there is finite sideway scattering. To show this, we plot the electric field patterns of the 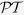-symmetric particle array at edge mode frequency in Fig. 5. The *x*- and *z*-components of the electric field are plotted in Fig. 5(a) and (b), respectively. It is observed that the distortion on the *E*_*x*_ component of the electric field is small, which is due to the fact that *E*_*x*_ mostly represents the light transmitted or reflected in the forward or backward directions.

In Fig. 4(b), we show the power spectrum absorbed/emitted by the mostly excited particle, which also represents the integrated electric field intensity in each particle. The dashed peak at *ω* = 0.5188*ω*_*p*_ for the case of *s* = 0.6*d* is produced by the simultaneously excited edge modes. A comparison with the case of *s* = 0.4*d* shows the significant contribution from the edge modes. This provides another piece of evidence that the edge modes are strongly excited even though forward extinction is vanishing.

Moreover, Fig. 5(b) shows that *E*_*z*_ is close to symmetric with reference to an inversion about the center of the array, which represents a nearly anti-symmetric response. Field patterns of the same particle array without gain/loss are also provided in Supplementary Information.

## Discussion

The anomalous scattering phenomenon demonstrated in dipole theory and rigorous multiple-multipole scattering theory in this paper suggests an extreme situation where the forward wave and backward scattered wave are nearly unaffected by the inclusion of the 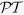-symmetric chain. If we consider a classical wave signal propagating in a waveguide or in free space, it might suggests that the scattered wave (i.e., the leakage of signal) can be unnoticeable to the transmitter and the receiver. If the incident wave carries some signals with small bandwidth within the bandgap, the receiver (who measures the transmitted wave) and the transmitter (who send the signals) will not see a difference before and after the 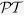-symmetric chain is inserted into the communication channel. This effect could suggest an approach to probe information in a two-way communication channel without affecting the original physical signals. It should be noted that this conclusion is made using purely classical theory and it may not be valid in the case where quantum effect is important. If that is the case, it may provide a method to demonstrate collective quantum effect.

In conclusion, we have predicted an anomalous light scattering property associated with the complex topological edge modes supported in a near-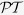-symmetric plasmonic system. Due to its special 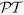-symmetric properties, such an anomalous scattering field is almost anti-symmetric when the system is excited by a symmetric plane wave. This resonantly scattered field has no contribution to the transmission and reflection parallel to the incident light, which means that the total forward extinction and feedback to the source is near zero. Since the resonant scattering is associated with the topological zero modes (which maps to a fixed frequency), the frequency at which the anomalous scattering occurs could be robust against weak perturbations. The result suggests an extreme situation where leakage of classical information is unnoticeable to the transmitter and the receiver. It should be noted that plasmonic particles are not the only candidates that support the anomalous scattering phenomenon. The main requirement to achieve such phenomenon is that the system is 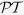-symmetric and edge modes are guaranteed to be in the 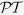-broken phase. The effect should be general for any 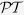-symmetric array of resonators with gain and loss, such as dielectric ring resonators.

## Methods

### Analytical evaluation of Zak phase

We evaluate the Zak phase analytically. The Zak phases discussed in all figures are evaluated using Eq. 3. The derivation of Eq. 3 (in usual basis) is provided here. For reference, the Zak phase in bi-orthonormal basis are also provided in Supplementary Information. To derive Eq. (4), we consider the eigenvalues and the right eigenvectors of Eq. (1), which are 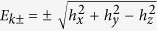 and


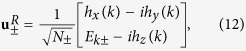


where the normalizing factor is defined as 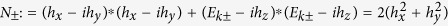, so that 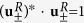. Although 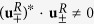, we still can evaluate the Zak phase of each band as those defined in Hermitian systems according to Eq. (3). We also provide another version of Zak phase defined in bi-orthonormal basis in Supplementary Information as a comparison.

Here, we parameterize the solution by putting 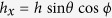, *h*_*y*_ = *h* sin *θ* sin *ϕ*, and *h*_*z*_ = *h*cos *θ*, where *θ* and *ϕ* are azimuthal and polar angle of 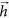
(*k*), and 
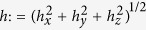
. As a result, the right eigenvectors in Eq. (12) are simplified as


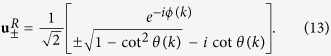


Using Eq. (13), we can write the integrant in the integral of Eq. (3) as 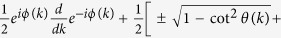

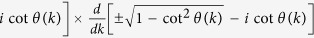
. Note that the condition 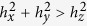 implies 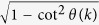 is real for all *k*, and by defining 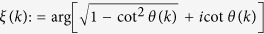, the second term can be simplified into 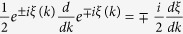. As a result, the Zak phase in Eq. (3) becomes


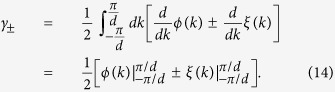


Since 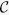 is closed, 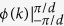 and 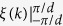 are integer multiples of 2*π*. Furthermore, since 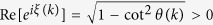 for all *k*, its argument is bounded (−*π*/2 < *ξ(k*) < *π*/2), which means 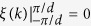. As a result, Eq. (14) gives Eq. (4).

### Coupled-dipole method

The particle array is modeled as an array of point dipole scatters embedded in air for the sake of simplicity. By the next nearest neighbor approximation together with quasistatic approximation, the dipole moment *p*_*n*;*σ*_ (*x* component) induced on the nanoparticle *σ* = *A* or *B* in the *n*th unit cell follows the coupled dipole equation^20^,^43^,^49^,^62^:


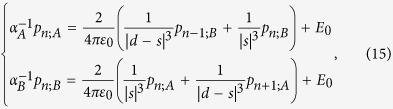


where *n* = 1, 2, …, *N* and *E*_0_ denotes the *x*-polarized external driving electric field.

Outer and inner radii of the coated nanoparticles are *b* and *a*, and the inverse quasistatic polarizabilites are given by^63^


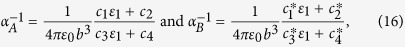


in which *c*_1_ = (*ε*_3_ + 2) + 2(*ε*_3_ − 1)(*a*/*b*)^3^, *c*_2_ = 2(*ε*_3_ + 2)*ε*_3_ − 2(*ε*_3_ − 1)*ε*_3_(*a*/*b*)^3^, *c*_3_ = (*ε*_3 _− 1) + (2*ε*_3_ + 1)(*a*/*b*)^3^, and *c*_4_ = 2*ε*_3_(*ε*_3 _− 1) + *ε*_3_(2*ε*_3_ + 1)(*a*/*b*)^3^. Note that *c*_*i*_ are constants that are independent of *ε*_1_.

### Formulation of eigenvalue problem for finite array

The eigenvalue problem in the form similar to Bergman’s representation is an important step to connect the actual problem to the topological band theory. Our formulation of eigenvalue problem is based on coupled-dipole method. It should be noted that obtaining resonant frequencies through this eigenvalue problem is much faster than searching complex non-trivial solutions directly using Eq. (15).

To do this, we group terms with *ε*_1_ in Eq. (15). The first and the second line are multiplied by *c*_3_*ε*_1_ + *c*_4_ and 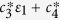 with respectively. By factoring out *ε*_1_, we have


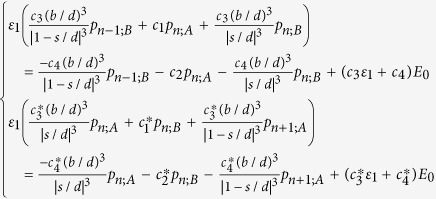


The above equations can be vectorized into the following matrix equation:


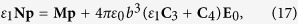


where 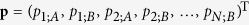 and **E**_0_ = (*E*_0_, *E*_0_, …. *E*_0_)^T^. The 2*N *× 2*N* matrixes are


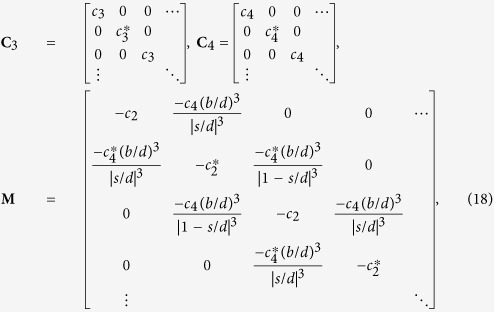



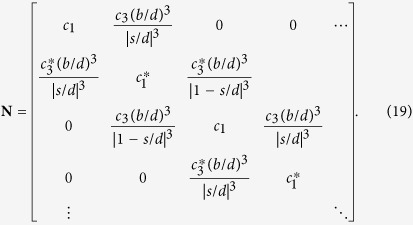


Multiplying Eq. (17) by *N*^−1^, we have


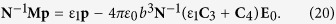


Rearranging, it becomes


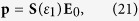


where **S**(ε_1_) = −4*πε*_0_*b*^3^(**N**^−1^**M **− ε_1_**I**_2*N*_)^−1^**N**^−1^(ε_1_**C**_3_ + **C**_4_). This recovers Eq. (8).

To look for resonant modes, we set E_0_ = 0 in Eq. (20), which gives an eigenvalue problem,


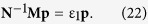


The 2*N* possible eigenvalues *ε*_1_ are mapped to *ω* by Eq. (6), giving 2*N* resonant frequencies, see Fig. 2(b); while the mode patterns are the 2*N* eigenvectors, see Fig. 2(c).

### Formulation of eigenvalue problem for infinite array

The formulation of the 2-band eigenvalue problem for an infinite array is again based on coupled-dipole method with the use of Bloch’s theorem. We first put *E*_0_ = 0 in Eq. (15). We write *p*_*n*;*A*_ = *p*_*k*;*A*_*e*^*iknd*^ and *p*_*n*;*B*_ = *p*_*k*;*B*_*e*^*iknd*^, and Eq. (15) becomes


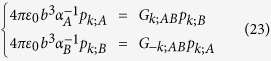


where *G*_*k*;*AB*_ = 2(*b*/*d*)^3^[*e*^−*ikd*^/(1 − *s*/*d*)^3^ + 1/(*s*/*d*)^3^]. To obtain an eigenvalue problem with *ε*_1_ as the eigenvalue, we group terms with *ε*_1_. Eq. (23) is then vectorized to the matrix equation ε_1_**N**_*k*_**p**_*k*_ = **M**_*k*_**p**_*k*_, in which **p**_*k*_ = (*p*_*k*;*A*_, *p*_*k*;*B*_)^T^,


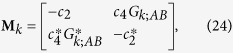


and


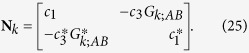


Multiplying the matrix equation by 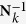, we recover the eigenvalue problem shown in Eq. (5):


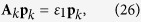


where


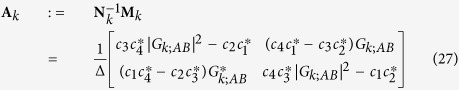


and 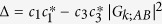 (which is always real). As *G*_*k*+2*π*/*d*;*AB*_ = *G*_*k*;*AB*_, we can write **A**_*k*_ = *f*_*k*_**I**_2_ + **H**_*k*_, where 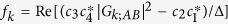, 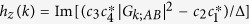,
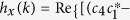

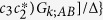
, 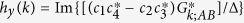, and **I**_2_ is a 2 × 2 identity matrix.

### Time reversal operator 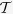


We define the time-reversal operator 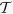 for the analysis of 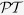 symmetry in the basis of coupled-dipole method. Suppose the time varying dipole moment on the *i*th nanoparticle has the general form 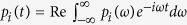, where *ω* is the angular frequency and *p*_*i*_(*ω*) is the Fourier component. Defining the time reverse operator by 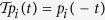, we have


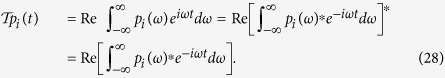


From the above, 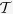 is thus turning the Fourier component into its complex conjugate, which means 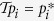.

For the case that the plasmonic cores are lossless, time reverse operation turns the energy gaining dielectric into energy losing dielectric and vice versa, which means the time reversed particle array is effectively the same as the particle array obtained by swapping the positions of nanoparticles *A* and *B*.

### Analysis of parity-time 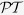 symmetry of matrix

The analysis of 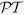 symmetry is again in the basis of coupled-dipole method. Here we would like to show that **S**(ε_1_) in Eq. (8) and 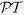 commute when 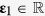.

Firstly, if a general 2*N* × 2*N* matrix *A* commutes with 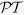, then *A*^−1^ commutes with 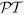 also. To show this, we consider the fact that **AA**^−1^ = **I**_2*N*_, we have 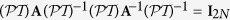. Since **A** commutes with 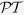 [i.e., 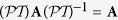], we have 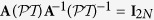. Further multiplying **A**^−1^ from the left, we have


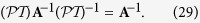


Secondly, if matrixes **A** and **B** commute with 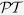, then their combination, **AB**, also commutes with 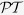:


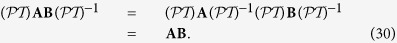


It is obvious to see from their definitions, **N**, **M**, **C**_3_, and **C**_4_ commute with 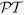. Thus, by the rules stated in Eqs (29) and (30), we have 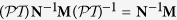. Extending the consideration gives


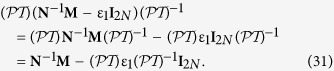


We see if *ε*_1_ is real, we have 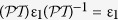, and therefore **N**^−1^**M **− ε_1_**I**_2*N*_ commutes with 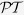. Similarly, ε_1_**C**_3_ + **C**_4_ commutes with 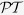 if ε_1_ is real. As a result, again by Eqs (29) and (30), recalling the definition of **S**(ε_1_) below Eq. (21), **S**(ε_1_) commutes with 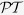 if ε_1_ is real.

### Analytical solutions of topological edge modes

The analytical solutions of the topological edge modes are based on coupled-dipole method in quasi-static approximation. For simplicity, we consider a semi-infinite plasmonic particle array with only one edge on the left. The protected edge modes are required to satisfy both the boundary condition and the dispersion relation with complex wave vectors *kd* = ±*π* + *iκd*^20^,^64^, where 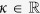.

By assuming the dipole moments are in the Bloch’s form with complex wave vectors, we have


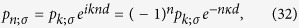


where *σ* = *A* or *B*. The dipole moments in the first two unit cells have to follow the coupled dipole equation at the boundary:


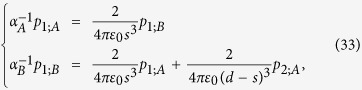


which is simply obtained by putting *p*_0;*σ*_ = 0 in Eq. (15). Substituting *p*_2;*A*_ = −*p*_1;*A*_*e*^*κd*^ to Eq. (33), and eliminating the fraction *p*_1;*B*_/*p*_1;*A*_, we have


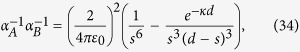


which is a condition obtained by considering the boundary. On the other hand, the existence of the nontrivial solution *p*_*k*;*σ*_ in Eq. (23) implies 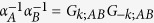. When *k* is real, it gives the dispersion relation; when *kd* = ± *π* + *κd*, it becomes


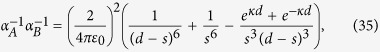


which is another condition for the edge state.

Eliminating 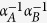 by Eqs (34) and (35), we obtain the decaying factor


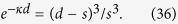


As only decaying dipole moments are physical (*κ* ≥ 0), there is no solution for *κ* unless *s* > 0.5*d*, which is the case with non-trivial Zak phase. By putting Eq. (36) back into Eq. (34), we have either 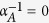 or 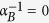. However, since 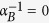 leads to the trivial solution *p*_1;*A*_ = *p*_1;*B*_ = 0 in Eq. (33), we have 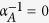. This implies ε_1_ = −*c*_2_/*c*_1_, *p*_1;*A*_ = 1, and *p*_1;*B*_ = 0. Putting back to the Drude model Eq. (6), we have


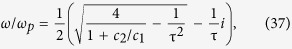


which is the left edge state resonant frequency. Solution for the particle array with a right edge can be obtained similarly. In this case, *e*^−*κd*^ = *s*^3^/(*d* − *s*)^3^ with *κ* < 0, and 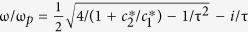.

### Multiple scattering theory

In our multiple scattering calculations, electromagnetic fields that satisfy the source free macroscopic Maxwell’s equations with the constitutive relations are expanded in series of vector spherical harmonics for each homogeneous region^60^,^61^ The dynamic multipoles (up to order of 5) of each coated particle coupled with all other particles through dynamic Green’s functions. The method is considered to be most accurate for spherical particles.

### Gauge freedom

There is a phase ambiguity on choosing eigenvectors in Eq. (12). One may choose another eigenvector 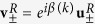, where *β(k*) is a real periodic function that *β(k* + 2*π*/*d*) = *β(k*) + 2*lπ* with integer *l*, and is known as the gauge freedom^38^,^39^. The Zak phase based on this gauge will be 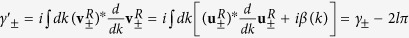. Therefore, due to this arbitrariness, the Zak phase is meaningful up to modulus 2*π*.

### Gain medium

There have been ways to realize the gain medium, for example, the use of semiconductor quantum dots^65^, dye molecules^66^,^67^, or rare-earth ions^68^. However, theoretical dispersive models on their effective permittivities are not unified, for example, they are modelled by Lorentizian feature^66^,^69^, assumed with 1/(*ω* − *ω*_0_) behaviour^70^, or just approximated by constants (non-dispersive)^5^,^71^.

## Additional Information

**How to cite this article**: Ling, C. W. *et al*. Anomalous Light Scattering by Topological 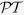-symmetric Particle Arrays. *Sci. Rep.* **6**, 38049; doi: 10.1038/srep38049 (2016).

**Publisher's note:** Springer Nature remains neutral with regard to jurisdictional claims in published maps and institutional affiliations.

## Supplementary Material

Supplementary Information
