# Supplementary material for: Anomalous Light Scattering by Topological PT-symmetric Particle Arrays
Source: Sci Rep. 2016 Dec 1;6:38049. doi: 10.1038/srep38049 (PMC5131320; doi:10.1038/srep38049)
Supplement: Supplementary Information [file srep38049-s1.pdf]

Anomalous Light Scattering by Topological $\mathcal{PT}$-symmetric Particle Arrays: Supplementary Information

C. W. Ling,[1] Ka Hei Choi,[1] T. C. Mok,[1] Z. Q. Zhang,[2] and Kin Hung Fung[1,*]

[1]*Department of Applied Physics, The Hong Kong Polytechnic University, Hong Kong, China*
[2]*Department of Physics, The Hong Kong University of Science and Technology, Hong Kong, China*

* khfung@polyu.edu.hk

A. ZAK PHASE AND BAND DISPERSION

In Fig. A.1(a), we show how the Zak phase γ (as defined in Eq. (3)) changes as the non-Hermiticity $\mathrm{Im}(\epsilon_3)$ increases. When $\mathrm{Im}(\epsilon_3) > 0.16$, BZ contains broken $\mathcal{PT}$-symmetric phase, and thus γ is not quantized. When $\mathrm{Im}(\epsilon_3) < 0.16$, bands with $s = 0.6d$ are non-trivial, which gives the protected edge modes (integral paths $\vec{h}(k)$ are shown in Fig. B.1. Bulk band dispersions with $\mathrm{Im}(\epsilon_3) = 0.025$ and 0.25 are demonstrated in Fig. A.1(b) for reference, and in which only (b)(ii) contains the exceptional points. Note that bulk dispersions for an array with $s = 0.4d$ and $s = 0.6d$ are the same, as the two infinite arrays only differ in a geometrical shift with $d/2$.

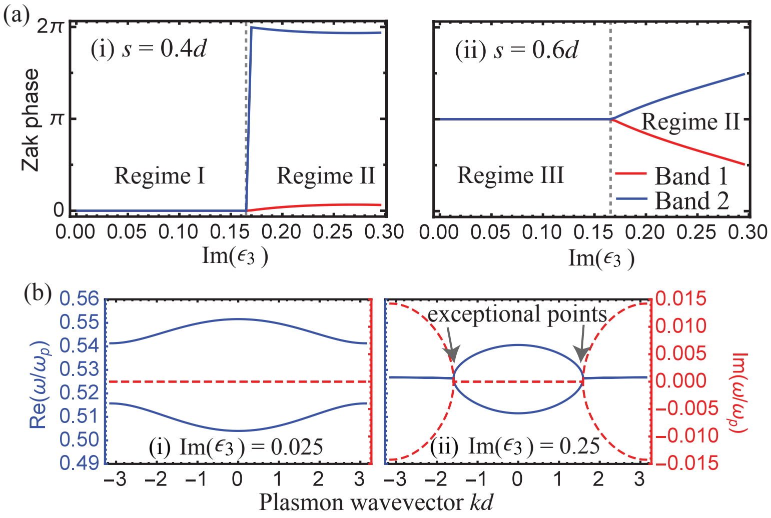

FIG. A.1. (Color online) (a) Zak phases γ with (i) $s = 0.4d$ and (ii) $s = 0.6d$. $\mathrm{Re}(\epsilon_3)$=1.5. When BZ contains broken $\mathcal{PT}$-symmetric phase (regime II), γ is not quantized. Otherwise, it is either classified as trivial (regime I, $\gamma = 0$) or non-trivial (regime II, $\gamma = \pi$). (b) Corresponding bulk dispersion relation for $s = 0.4d$ or $s = 0.6d$ when (i) $\mathrm{Im}(\epsilon_3) = 0.025$ and (ii) $\mathrm{Im}(\epsilon_3) = 0.25$.

B. EXACT $\vec{h}$-SPACE DIAGRAM FOR PLASMONIC DIMER ARRAYS

A closed loop is formed by $\vec{h}(k)$ when kd changes from $-\pi$ to π, as shown in Fig. B.1. Here, $\mathbf{H}_k$ has the form of Eq. (12). Since the additional term $f_k \mathbf{I}_2$ does not alter the eigenvectors, $\mathbf{A}_k$ share the same eigenvectors and Zak phase γ with $\mathbf{H}_k$. The two eigenvalues are mapped to ω via Eq. (6), which gives the dispersion relation in Fig. A.1(b).

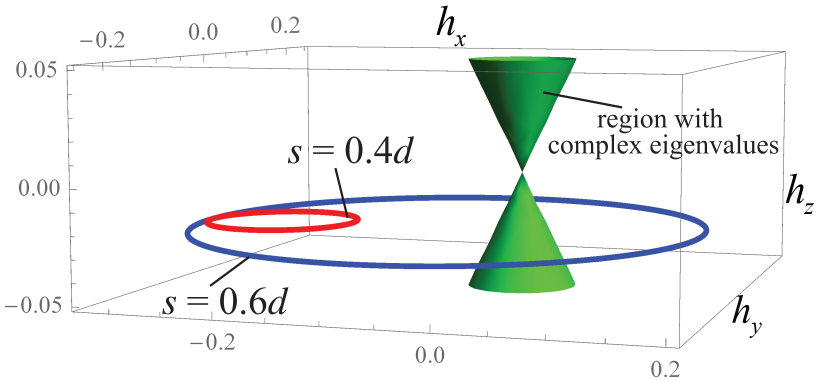

FIG. B.1. (Color online) Numerical integral path $\vec{h}(k)$ of an infinite plasmonic particle array. Parameters are $a = 0.125d$, $b = 0.175d$, $1/\tau = 0$, $\epsilon_3 = 1.5 + 0.025i$. The region within the kissing cones is where eigenvalues are complex, which corresponds to the broken $\mathcal{PT}$-symmetric phase.

C. ZAK PHASE WITH BI-ORTHONORMAL BASIS

If bi-orthonormal bases are used to define γ instead of the usual definition, γ will be in general a complex number. However, its real part gives back the value obtained by Eq. (3). Here, we provide the evaluation of Zak phase based on the bi-orthornormal basis.

Recalling $\mathbf{H}_k$ in Eq. (1) is non-Hermitian, therefore, left eigenvectors have to be used to form a bi-orthonormal basis. The left eigenvector $\mathbf{u}^L$ satisfies the eigenvalue problem $\mathbf{H}_k^{\mathrm{T}}\mathbf{u}^L = E_k\mathbf{u}^L$ [1–4], whose eigenvalues are $E_{k\pm} = \pm(h_\parallel^2 - h_z^2)^{1/2}$, where $h_\parallel = (h_x^2 + h_y^2)^{1/2}$. The right and left eigenvectors of $\mathbf{H}_k$ are

$$\mathbf{u}_\pm^R = \frac{1}{{M_{k\pm}}^{1/2}}\begin{bmatrix} h_x(k) - ih_y(k) \\ E_{k\pm} - ih_z(k)\end{bmatrix} \tag{C.1a}$$

and

$$\mathbf{u}_\pm^L = \frac{1}{{M_{k\pm}}^{1/2}}\begin{bmatrix} h_x(k) + ih_y(k) \\ E_{k\pm} - ih_z(k)\end{bmatrix}, \tag{C.1b}$$

where the normalizing factor $M_{k\pm} := (h_x - ih_y)(h_x + ih_y) + (E_{k\pm} - ih_z)^2 = h_\parallel^2 + (E_{k\pm} - ih_z)^2 = 2h_\parallel^2 - 2h_z^2 \pm 2ih_z\sqrt{h_\parallel^2 - h_z^2}$. We note that $M_{k\pm}$ is chosen such that the biorthnormal conditions $\mathbf{u}_\pm^L \cdot \mathbf{u}_\pm^R = 1$ and $\mathbf{u}_\mp^L \cdot \mathbf{u}_\pm^R = 0$ are satisfied.

The Zak phase defined by using biorthonormal basis is [1, 2, 4]

$$\gamma_\pm = i\int_{-\frac{\pi}{d}}^{\frac{\pi}{d}} dk\left(\mathbf{u}_\pm^L \cdot \frac{d}{dk}\mathbf{u}_\pm^R\right), \tag{C.2}$$

which is similar to Eq. (3). In this case, $\gamma_\pm$ will be a complex number even the entire bulk dispersion is in the unbroken $\mathcal{PT}$-symmetric phase, and its real part is the same as that in Eq. (4). To show this, we again restrict $E_{k\pm}$ are real. We put $h_x(k) + ih_y(k) = h_\parallel(k)e^{i\phi(k)}$. Using the product rule, Eq. (C.2) becomes

$$\begin{aligned}\gamma_\pm =& i\int_{-\frac{\pi}{d}}^{\frac{\pi}{d}} dk\left(\frac{h_\parallel e^{i\phi}}{M_{k\pm}}\frac{d}{dk}h_\parallel e^{-i\phi}\right.\\ &+ \frac{h_\parallel^2}{{M_{k\pm}}^{1/2}}\frac{d}{dk}\frac{1}{{M_{k\pm}}^{1/2}} + \frac{E_{k\pm} - ih_z}{M_{k\pm}}\frac{d}{dk}\left(E_{k\pm} - ih_z\right)\\ &\left.+ \frac{\left(E_{k\pm} - ih_z\right)^2}{{M_{k\pm}}^{1/2}}\frac{d}{dk}\frac{1}{{M_{k\pm}}^{1/2}}\right).\end{aligned} \tag{C.3}$$

The first term in the integrant gives

$$\begin{aligned}\frac{h_\parallel e^{i\phi}}{M_{k\pm}}\frac{d}{dk}h_\parallel e^{-i\phi} &= \frac{-ih_\parallel^2}{M_{k\pm}}\frac{d}{dk}\phi + \frac{h_\parallel}{M_{k\pm}}\frac{d}{dk}h_\parallel\\ &= \frac{-ih_\parallel^2}{M_{k\pm}}\frac{d}{dk}\phi + \frac{1}{2M_{k\pm}}\frac{d}{dk}h_\parallel^2.\end{aligned} \tag{C.4a}$$

The second and the forth terms give

$$\begin{aligned}&\frac{h_\parallel^2}{{M_{k\pm}}^{1/2}}\frac{d}{dk}\frac{1}{{M_{k\pm}}^{1/2}} + \frac{\left(E_{k\pm} - ih_z\right)^2}{{M_{k\pm}}^{1/2}}\frac{d}{dk}\frac{1}{{M_{k\pm}}^{1/2}}\\ &= \frac{h_\parallel^2 + \left(E_{k\pm} - ih_z\right)^2}{{M_{k\pm}}^{1/2}}\frac{d}{dk}\frac{1}{{M_{k\pm}}^{1/2}}\\ &= {M_{k\pm}}^{1/2}\frac{d}{dk}\frac{1}{{M_{k\pm}}^{1/2}} = -\frac{1}{2M_{k\pm}}\frac{d}{dk}M_{k\pm},\end{aligned} \tag{C.4b}$$

in which we used the definition of $M_{k\pm}$ [below Eq. (C.1)]. The third term gives

$$\frac{E_{k\pm} - ih_z}{M_{k\pm}}\frac{d}{dk}\left(E_{k\pm} - ih_z\right) = \frac{1}{2M_{k\pm}}\frac{d}{dk}\left(E_{k\pm} - ih_z\right)^2. \tag{C.4c}$$

Substituting Eqs. (C.4) into Eq. (C.3), we have

$$\begin{aligned}\gamma_{\pm} =& i\int_{-\frac{\pi}{d}}^{\frac{\pi}{d}} dk\left(\frac{-ih_{\parallel}^2}{M_{k\pm}}\frac{d}{dk}\phi+\frac{1}{2M_{k\pm}}\frac{d}{dk}h_{\parallel}^2\right.\\ &\left.-\frac{1}{2M_{k\pm}}\frac{d}{dk}M_{k\pm}+\frac{1}{2M_{k\pm}}\frac{d}{dk}\left(E_{k\pm}-ih_z\right)^2\right)\\ =& i\int_{-\frac{\pi}{d}}^{\frac{\pi}{d}} dk\left(\frac{-ih_{\parallel}^2}{M_{k\pm}}\frac{d}{dk}\phi+\frac{1}{2M_{k\pm}}\frac{d}{dk}M_{k\pm}\right.\\ &\left.-\frac{1}{2M_{k\pm}}\frac{d}{dk}M_{k\pm}\right)=\int_{-\frac{\pi}{d}}^{\frac{\pi}{d}} dk\frac{h_{\parallel}^2}{M_{k\pm}}\frac{d}{dk}\phi.\end{aligned} \tag{C.5}$$

Noticing that as long as $E_{k\pm}$ are real, $(h_{\parallel}^2-h_z^2)^{1/2}$ are also real. Then, by rationalizing the fraction in the last line of Eq. (C.5), we have

$$\begin{aligned}\frac{h_{\parallel}^2}{M_{k\pm}} &= \frac{1}{2}\frac{h_{\parallel}^2}{h_{\parallel}^2-h_z^2\pm ih_{\parallel}^2h_z(h_{\parallel}^2-h_z^2)^{1/2}}\\ &= \frac{1}{2}\frac{h_{\parallel}^2\mp ih_{\parallel}^2h_z/(h_{\parallel}^2-h_z^2)^{1/2}}{h_{\parallel}^2}\\ &= \frac{1}{2}\mp i\frac{h_z}{2(h_{\parallel}^2-h_z^2)^{1/2}}.\end{aligned} \tag{C.6}$$

Finally, by putting Eq. (C.6) into Eq. (C.5), we have

$$\begin{aligned}\gamma_{\pm} &= \int_{-\pi/d}^{\pi/d}\frac{d\phi}{dk}dk\mp i\int_{-\pi/d}^{\pi/d}\frac{h_z}{2(h_{\parallel}^2-h_z^2)^{1/2}}dk\\ &= w\pi\mp i\int_{-\pi/d}^{\pi/d}\frac{h_z}{2(h_{\parallel}^2-h_z^2)^{1/2}}dk,\end{aligned} \tag{C.7}$$

where w is the winding number of $\vec{h}(k)$ about the h_z axis. Eq. (C.7) shows that γ is in general a complex number, unless $\vec{h}(k)$ has some other symmetries so that the integral vanishes.

D. FIELD PATTERNS FOR A NORMAL ARRAY ($\epsilon_3 = 1.5$) BY MST

For comparison with Fig. 5(b), here we show the electric field pattern of a normal array with $\epsilon_3 = 1.5$ in Fig. D.1. Since the normal array $\mathcal{P}$ symmetry, the response of the array should be symmetric, which gives an anti-symmetric pattern for the E_z field component. The two figures (Figs. 5 and D.1) verified the strong antisymmetry response of the non-Hermitian particle array, which does not contribute to forward and backward scattering.

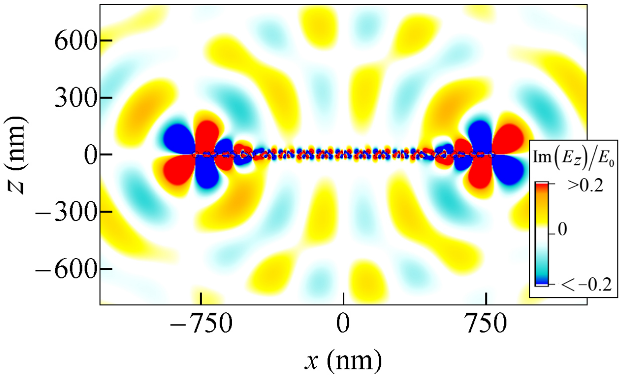

FIG. D.1. (Color online) Electric field pattern of the E_z field component at edge mode frequency ($\omega = 0.5188\omega_p$) of the normal array (with $\epsilon_3 = 1.5$ and $s = 0.6d$).

[1] J. C. Garrison and E. M. Wright, Complex Geometrical Phases for Dissipative Systems, Phys. Lett. A **128**, 177 (1988).
[2] A. I. Nesterov and F. A. de la Cruz, Complex magnetic monopoles, geometric phases and quantum evolution in the vicinity of diabolic and exceptional points, J. Phys. A: Math. Theor. **41**, 485304 (2008).
[3] P. Lancaster and M. Tismenetsky, The Theory of Matrices, chap. 4.10, 2nd ed. (Academic Press Inc., Orlando, Florida 32887, 1985).
[4] A. A. Mailybaev, O. N. Kirillov, and A. P. Seyranian, Geometric phase around exceptional points, Phys. Rev. A **72**, 014104 (2005).
